# On the Behavior of Plaster of Paris in Setting

**Published:** 1881-01

**Authors:** W. Bowman Macleod


### ON THE BEHAVIOR OF PLASTER OF PARIS IN SETTING.

BY W. BOWMAN MACLEOD, L. D. S., EDIN.

*From the Transactions of the Odonto-Chirurgical Society.*

The subject of the communication which I have now the honor of laying before this Society is, "On the Behavior of Plaster of Paris in Setting." Its intention is to bring under your notice the results of several experiments entered into for the purpose of demonstrating, by practical examples, to my class, the minute amount of expansion which takes place during the setting of plaster of Paris, such as we ordinarily use in the laboratory, and which resulted — in so far as I was concerned — in drawing my attention to another characteristic which has hitherto been unsuspected, and if suspected, not sufficiently acknowledged as a factor in dental mechanics. The presence of this peculiarity has, I believe, in many cases, produced faults in modeling and fitting which have been attributed to other causes; and a remedy having been sought for in the wrong direction, has not hitherto been found. I refer to the rocking of plates upon the middle line of the palatine arch, the general misfit of plates, and the opening of the joints in gum blockwork. To prove that plaster expanded, I cast a quantity of it within a square of two feet, the sides of which were enclosed with iron plates three-quarters of an inch in depth, and closely fitted together but unattached, supported by angle ties, and retained in position by a piece of cord tied round their outer circumference. The plaster was cast within this area, and as it set sufficiently to hold itself together, the cord was cut, and the mass allowed to crystallize without being bound laterally. On measuring this block the following day, I found that it had increased by five sixteenths of an inch in length, and the same in breadth. This being reduced proportionally to the average breadth of the dental arch, would certainly have made very little difference, practically, in the fitting in the majority of cases, being only the one-thirtyseventh part of an inch of expansion on the average denture. But I found that not only had the plaster expanded, but the upper surface was raised; and on sawing the block through in a diagonal direction, I found that instead of the block lying dead upon the plane beneath, it presented a concave surface toward the plane, the highest point of which measured one-half inch. This showed, first of all, that the plaster had not only expanded, but had done something more than its now greater length and breadth would lead one to suspect; for in thus taking a concave form, it must have either retracted to an equal extent, or expanded in an irregular manner, causing warpage.

Making still further experiments by casting plaster in the ordinary impression cup, I found, invariably, the same result produced, and that the centre portion — the palatine portion — of the cup always presented an open and well defined space between the upper surface of the impression cup and the lower surface of the hardened plaster. This circumstance, therefore, would produce in your model a fault similar to that resulting from the sucking of the waxy or resinous impression materials, and, as you can readily see, would give you a much higher dome than that of the natural arch. Hence the rocking of the plate, which has hitherto been attributed — if my deductions be justified — to every cause but the right one. Continuing my researches, I found that although in the equal surface and depth of the modeling tray, the defect always ran in one direction, yet on pouring the plaster into irregular moulds, such as the impression of the mouth, the position of the point of warpage was not always persistent, but seemed to be controlled by the thickness of the superincumbent layer of plaster, and this led to the conclusion that while in some instances the defect would determine itself on the palatine ridge in the shape of an exaggerated dome, at other times, and that more frequently in under dentures, it would express itself in irregular lateral expansion, and consequent misfit upon the posterior portion of the alveolar ridge. Naturally, then, I began to inquire how this defect might be overcome, and I find that by the addition to the water with which the plaster is mixed of potash alum (hitherto used entirely for the purpose of quick setting in impression taking), in the proportion of from three to four ounces to the gallon, you will entirely overcome the irregular expansion and consequent warping which takes place in coarse plaster of Paris as used with water alone. But here you have the two blocks of equal dimensions — one cast with water, and the other with potash alum water. It requires no explanation on my part to point out the difference between the two. In the one case, the expansion is five-sixteenths of an inch; warpage, one-half inch. In the other, expansion, *nil;* and warpage, ditto; and the two surfaces, dead. You have here a series of impression cups of various sizes and shapes, filled with plaster, cast with pure or plain water, and with potash alum, and which require but to be examined to convince you of the fact of the deadness of plaster of Paris when treated with potash alum, and its behavior under ordinary circumstances. The conclusion I draw from this is, that all plaster, either for impression taking or for models, should be cast with potash alum, when strict and definite results are to be obtained; and that in the case of gum blockwork, that opening of the joints — which has hitherto caused so much trouble to practitioners, and, to a great exent, has prevented the more general adoption its other merits might have commanded, and which has drawn out many suggestions as to the best mode of prevention — the opening of the joints may now be entirely prevented by the use of potash alum for both matrix and model within the flask.

[Solution of Potash Alum for modeling in plaster of Paris has been used by many for years, but the experiments recorded in the foregoing paper justify us in reproducing it here. Alum of commerce, until late years, was prepared according to the following formula: — $Al_2\ 3\ S\ O_4\ Am_2\ S\ O_4\ 24\ H_2O$. Manufacturing chemists are now frequently using $Al_2\ 3\ S\ O_4\ K_2\ S\ O_4\ 24\ H_2O$.— Ed. *M. R D. S.*]

Inhalation of Oxygen Gas is reported as having succeeded in opium poisoning after the failure of the ordinary remedies.

A Bogus Diploma mill has been found in Boston, the title of which is "The Nova Anglica Universatis Scientiæ et Artium."

